# Machine Learning-Based Model for Predicting Incidence and Severity of Acute Ischemic Stroke in Anterior Circulation Large Vessel Occlusion

**DOI:** 10.3389/fneur.2021.749599

**Published:** 2021-12-02

**Authors:** Junzhao Cui, Jingyi Yang, Kun Zhang, Guodong Xu, Ruijie Zhao, Xipeng Li, Luji Liu, Yipu Zhu, Lixia Zhou, Ping Yu, Lei Xu, Tong Li, Jing Tian, Pandi Zhao, Si Yuan, Qisong Wang, Li Guo, Xiaoyun Liu

**Affiliations:** ^1^Department of Neurology, The Second Hospital of Hebei Medical University, Shijiazhuang, China; ^2^Department of Information Center, The Second Hospital of Hebei Medical University, Shijiazhuang, China; ^3^Department of Neurology, Hebei Province People's Hospital, Shijiazhuang, China; ^4^Department of Neurology, Xingtai People's Hospital, Xingtai, China; ^5^Department of Medical Iconography, The Second Hospital of Hebei Medical University, Shijiazhuang, China; ^6^Neuroscience Research Center, Medicine and Health Institute, Hebei Medical University, Shijiazhuang, China

**Keywords:** anterior circulation large vessel occlusion, acute ischemic stroke, machine learning, prediction model, neurological impairment

## Abstract

**Objectives:** Patients with anterior circulation large vessel occlusion are at high risk of acute ischemic stroke, which could be disabling or fatal. In this study, we applied machine learning to develop and validate two prediction models for acute ischemic stroke (Model 1) and severity of neurological impairment (Model 2), both caused by anterior circulation large vessel occlusion (AC-LVO), based on medical history and neuroimaging data of patients on admission.

**Methods:** A total of 1,100 patients with AC- LVO from the Second Hospital of Hebei Medical University in North China were enrolled, of which 713 patients presented with acute ischemic stroke (AIS) related to AC- LVO and 387 presented with the non-acute ischemic cerebrovascular event. Among patients with the non-acute ischemic cerebrovascular events, 173 with prior stroke or TIA were excluded. Finally, 927 patients with AC-LVO were entered into the derivation cohort. In the external validation cohort, 150 patients with AC-LVO from the Hebei Province People's Hospital, including 99 patients with AIS related to AC- LVO and 51 asymptomatic AC-LVO patients, were retrospectively reviewed. We developed four machine learning models [logistic regression (LR), regularized LR (RLR), support vector machine (SVM), and random forest (RF)], whose performance was internally validated using 5-fold cross-validation. The performance of each machine learning model for the area under the receiver operating characteristic curve (ROC-AUC) was compared and the variables of each algorithm were ranked.

**Results:** In model 1, among the included patients with AC-LVO, 713 (76.9%) and 99 (66%) suffered an acute ischemic stroke in the derivation and external validation cohorts, respectively. The ROC-AUC of LR, RLR and SVM were significantly higher than that of the RF in the external validation cohorts [0.66 (95% CI 0.57–0.74) for LR, 0.66 (95% CI 0.57–0.74) for RLR, 0.55 (95% CI 0.45–0.64) for RF and 0.67 (95% CI 0.58–0.76) for SVM]. In model 2, 254 (53.9%) and 31 (37.8%) patients suffered disabling ischemic stroke in the derivation and external validation cohorts, respectively. There was no difference in AUC among the four machine learning algorithms in the external validation cohorts.

**Conclusions:** Machine learning methods with multiple clinical variables have the ability to predict acute ischemic stroke and the severity of neurological impairment in patients with AC-LVO.

## Introduction

Acute ischemic stroke caused by large vessel occlusion accounts for more than 40% of cases, ~80% of which occurs in the anterior circulation (1). Compared to non-large vessel occlusion (LVO) acute ischemic stroke (AIS), patients with anterior circulation large vessel occlusion (AC-LVO) stroke are considered to be at greater risk of mortality or disability before endovascular treatment (2). They tend to improve significantly after mechanical thrombectomy (3, 4). Previously reported prediction models for AC-LVO stroke such as prehospital scales (Prehospital Acute Stroke Severity scale, PASS; Cincinnati Prehospital Stroke Severity Scale, CPSSS; stroke Vision Aphasia Neglect, VAN; Rapid Arterial Occlusion Evaluation scale RACE and Field Assessment Stroke Tri-age for Emergency Destination, FAST-ED) (5–9) that are based on NIHSS, and the recently proposed model by Philipp Hendrix et al., which combines past medical history and neurologic examination (10), have focused on the identification of large vessel occlusion in patients with AIS. The main clinical purpose of the prediction scores is to identify which patients with AIS have LVO so that they can be referred to capable centers for endovascular treatment (EVT). However, accurate prediction of AIS in patients with AC-LVO remains a challenge.

Anterior circulation-LVO stroke can be further divided based on pathogenesis and severity of clinical consequences, into non-disabling and disabling stroke with the latter frequently resulting in post-stroke dependence. Nevertheless, no previous studies have predicted the risk of disabling ischemic stroke in patients with AC-LVO, which may be useful in treatment decisions and prevention.

In this study, we developed and validated two models based on machine learning algorithms with clinical variables, to predict acute ischemic stroke (Model 1) and severity of neurological impairment (Model 2) in patients with AC-LVO.

## Methods

### Patient Cohorts

A total of 1,100 patients with AC- LVO admitted between June 2016 and April 2018 at the Second Hospital of Hebei Medical University, North China, were registered in the derivation cohort; 927 of them who presented with AIS related with AC-LVO and asymptomatic AC-LVO were retrospectively reviewed. In addition, 471 patients with first-ever ischemic stroke (including disabling and non-disabling stroke) were selected. For the external validation, we collected data of patients with AC-LVO from Hebei Province People's Hospital, China between September 2016 and April 2021.

Anterior circulation-LVO was defined as complete occlusion of at least one intracranial internal carotid artery (ICA) or middle cerebral artery (MCA) visualized on computed tomography angiography (CTA) or magnetic resonance angiography (MRA). ICA occlusion refers to the complete occlusion of the C1–C7 segment of the internal carotid artery based on CTA or MRA. MCA occlusion refers to the occlusion of the MCA involving at least the M1 segment (for more details please see in Supplementary Figure I). Asymptomatic AC-LVO was defined as the absence of a transient ischemic attack (TIA), amaurosis fugax, and ischemic stroke attributed to anterior circulation large vessel (11, 12). In accordance with previous studies, disabling and non-disabling ischemic strokes were defined by the initial clinician as National Institutes of Health Stroke Scale (NIHSS) > 5 and ≤ 5 on admission, respectively (13).

### Data Collection and Variable Selection

Patient characteristics that were collected on admission for the development of Models 1 and 2 include (1) demographic data of the patients such as the age, sex, body mass index (BMI), current smoking and drinking status, comorbidity (hypertension, coronary atherosclerotic heart disease, atrial fibrillation, diabetes mellitus, and hyperlipidemia), history of transient ischemic attack (TIA); (2) clinical variables such as serum apolipoprotein B (Apo B) and homocysteine on arrival; (3) imaging variables such as occluded vessels (unilateral MCA, unilateral ICA, and multiple arteries), posterior circulation large vessel severe stenosis (≥ 70%) /occlusion, anterior cerebral artery (ACA) occlusion, and Alberta Stroke Program Early CT Score (ASPECTS). Data on 14 variables were included in Model 1, and on 12 in Model 2. Specifically, normal blood flow status of the vertebrobasilar arteries *via* the posterior communicating artery plays a major role in primary collateral compensation after anterior circulation large vessel occlusion. Therefore, posterior circulation large vessel stenosis/occlusion was introduced into Model 1. Posterior circulation large vessel refers to the intracranial vertebral artery, basilar artery, or segment P1 of the posterior cerebral artery.

### Data Pre-processing

Processing of the data was performed using Python. First, records containing outliers, which were identified by boxplot, were excluded. Furthermore, the median imputation method was used to impute missing values in derivation cohorts. Finally, the categorical variables were converted into numerical values with dummy encoding, and the continuous features were standardized by removing the mean and scaling to unit variance.

### Prediction Models With Machine Learning

Machine learning is a discipline that constructs models base on data, which is a part of artificial intelligence. Machine learning extracts the characteristics and abstracts the model of the data, discovers the information in the data, and then analyzes and predicts it. First, an algorithm and some parameters of the model which were supplied with training data were selected arbitrarily. During training procedures, the model automatically adjusts some trainable parameters stage by stage to achieve better performance optimization. After the training, all the model parameters are fixed. Importantly, the true effectiveness of the model was evaluated using test data that were completely separate from the training data.

We selected logistic regression without regularization (LR), regularized logistic regression (RLR), random forest (RF), and support vector machine (SVM) as machine learning algorithms that are commonly used.

Logistic regression, a classic classification algorithm in machine learning, was regularized using a combination of L1 and L2 loss in this study. Here, the target was determined by Y:


$$\begin{array}{r} \text{Y}=\{“\text{disabling ischemic stroke}{,}^{\prime \prime }\\ “\text{ non-disabling ischemic stroke}\prime \prime \}\\ \text{Z}={\text{W}}^{\text{T}}\text{X}+\text{b}\\ \text{y}=\frac{1}{1+{\text{e}}^{-\text{Z}}}=\frac{1}{1+{\text{e}}^{{\text{-W}}^{\text{T}}\text{X+b}}} \end{array}$$

We selected binary cross-entropy loss as the cost function, where y is the ground truth, y^∧^ is the predicted score of the model, and R represents the regularization. The loss functions L1 and L2 are defined as follows:


$$\begin{array}{r} \text{J}\left(\text{w},\text{b}\right)\text{=}\frac{\text{1}}{\text{m}}\sum_{\text{i=1}}^{\text{m}}\text{L}\left(\hat{{\text{y}}^{\left(\text{i}\right)}},{\text{y}}^{\left(\text{i}\right)}\right)\\ =\frac{\text{1}}{\text{m}}\sum_{\text{i=1}}^{\text{m}}\left({\text{-y}}^{\left(\text{i}\right)}\log\hat{{\text{y}}^{\left(\text{i}\right)}}\text{-}\left({\text{1-y}}^{\left(\text{i}\right)}\right)\log\left(\text{1-}\hat{{\text{y}}^{\left(\text{i}\right)}}\right)\right)\text{+}\frac{\lambda }{\text{2}}\text{R}\\ {\text{R}}_{\text{L1}}\text{=}\sum_{\text{j=1}}^{\text{m}}\left|{\text{W}}_{\text{j}}\right|....{\text{R}}_{\text{L2}}\text{=}\sum_{\text{j=1}}^{\text{m}}\left[{\text{W}}_{\text{j}}^{\text{2}}\right] \end{array}$$

In the training process of the model, standardization of numerical variables was carried out to accelerate the convergence process and speed of the model.

Random forest is an extended variant of bagging, which uses a decision tree as the base learner and introduces the selection of random attributes in the training process of the decision tree (14). The main parameters that can affect the model performance in RF include the number of trees in the forest, maximum depth of the tree, minimum number of samples required to split an internal node, minimum number of samples required to be at a leaf node, and function to measure the quality of a split. In this study, the values in the dataset were discretized, and the parameters were optimized with a grid search during the training process.

An SVM classifies data by calculating the maximum-margin hyperplane, which adds a regularization term in the solving process to optimize the structural risk. The strength of SVM is that it can process complex datasets with many variables or dimensions (15). The validity of SVM depends mainly on the selection of the kernel function, parameters of the kernel, and soft margin parameter C. Otherwise, in this study, each combination of parameter selections was checked using cross-validation, and only parameters with optimal accuracy were selected.

Moreover, LR, RLR, RF, and SVM can estimate the contribution of each feature to the model by calculating the absolute value of the standardized regression coefficient, information gain / Gini coefficient, and weight coefficient.

### Model Derivation and Internal Validation

**Figure 1:**
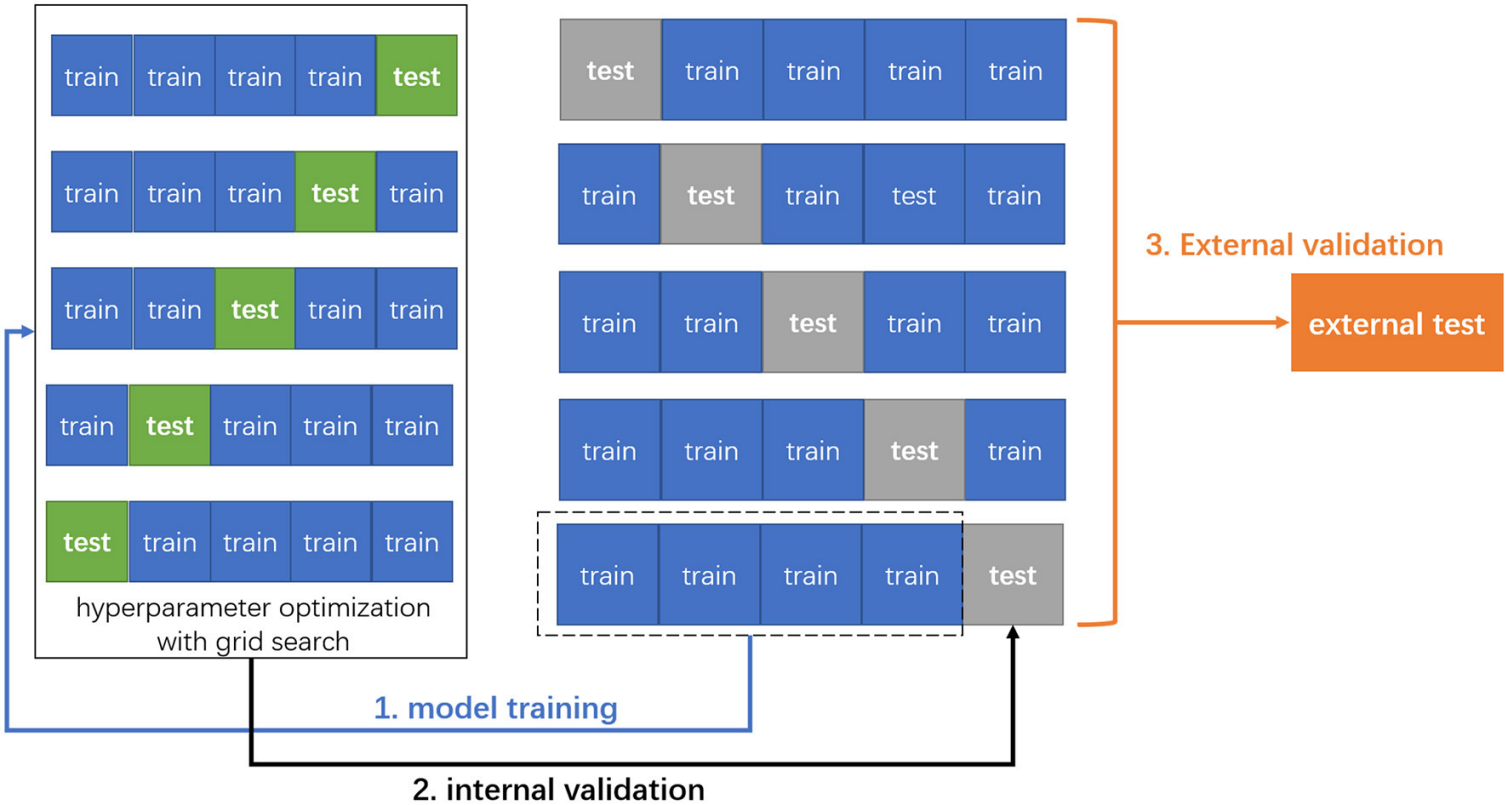
The schema of 5-fold cross-validation and external validation. First, the original data were randomly divided into five patterns without duplication, one of which was used as the test set, and the remaining four as the training set for model training. Next, we adapted grid search with 5-fold cross-validation to optimize the hyperparameters for each machine learning model. Finally, the trained models were externally validated on the external test data.

In this study, for model derivation, we adopted 5-fold cross-validation, which is a standard way of optimizing the model with inner test data and has been used in a previous study (16). During modeling, the grid search algorithm which is a greedy algorithm was combined to tune and optimize the model hyperparameters. For each group of hyperparameters, we selected 5-fold cross-validation to determine the optimal ones, after which we calculated the means of sensitivity, specificity, accuracy, and AUC to evaluate the performance of each model (Figure 1).

The derivation and validation models were conducted using Python 3.6. The model algorithms, cross-validation, and grid search were based on the Scikit-Learn library of Python in the PyCharm. Matplotlib 3.3.3, NumPy 1.19.5, pandas version 1.1.5, and Scikit-Learn toolkit version 0.21.0 were used to train the machine learning models.

### External Validation

After internal training and testing, the performance of the model was evaluated using external validation data. Subsequently, the AUCs were compared among machine learning algorithms.

### Statistical Analysis

Clinical variables are presented as mean ± *SD* or median with interquartile range, depending on the distribution of the variables. To compare the group differences, continuous variables were compared using the Student's *t*-test or Mann-Whitney *U* test, and categorical variables were compared with the χ2 test or Fisher's exact test. These two prediction models were discriminated against using AUC. Calculation of AUC, sensitivity, specificity, precision, negative predictive value (NPV), and accuracy criteria were performed with R statistical software version 3.0.2. The area under the precision-recall curve (PRC) and F1-score were calculated with MedCalc. For the derivation and validation cohorts, a comparison of AUC among the machine learning methods was performed using the DeLong test with Bonferroni correction. Two-sided *P* < 0.05 were considered statistically significant.

## Results

### Baseline Characteristics

**Figure 2:**
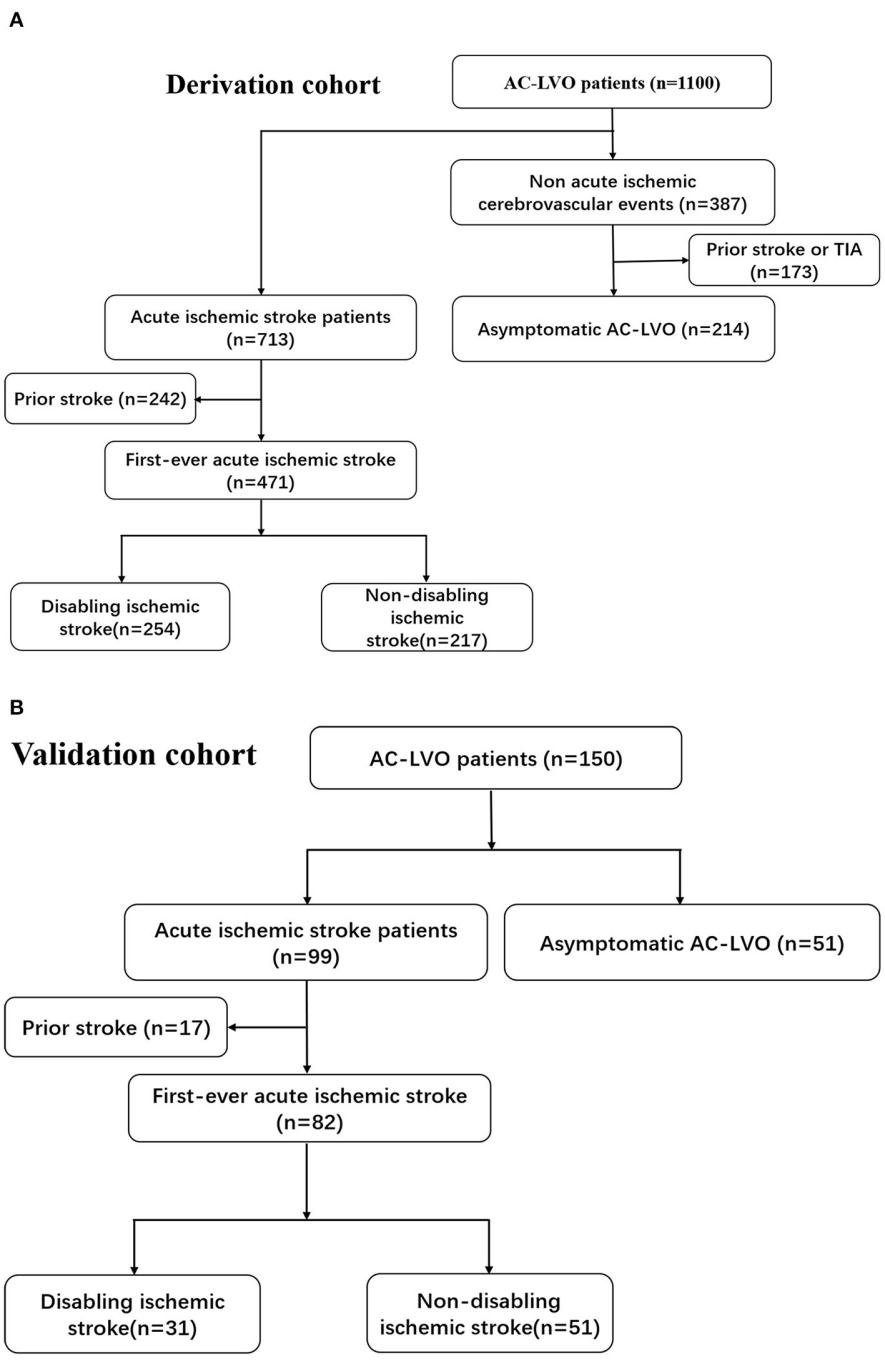
The flow diagram of the patients included in this study is shown in **(A,B)**.

Figures 2A,B illustrate the flow diagram of the enrolled patients. For the derivation cohort, 1,100 patients with AC-LVO were hospitalized at the study institution. After excluding 173 patients with prior stroke or TIA as non-acute ischemic cerebrovascular events, 713 patients with AIS related with AC-LAO and 214 with asymptomatic AC-LAO were finally included in the analysis for model 1. Among the 214 patients with asymptomatic AC-LVO, 119 (56%) were hospitalized for head discomfort such as heaviness of the head and fullness in the head. The other reasons for hospitalization in patients with asymptomatic AC-LVO included coronary artery disease, subarachnoid hemorrhage, migraine, cerebral large artery disease detected by routine physical examination, unruptured intracranial aneurysms, diabetic peripheral vascular disease, central nervous system infection, lower extremity atherosclerotic occlusive disease, intracranial space-occupying lesions, epilepsy, Parkinson's disease, cerebral atrophy, subclavian artery steal blood syndrome, cough syncope, and cardiac syncope. The general screening of large artery disease was performed with transcranial Doppler and carotid artery ultrasound in these patients. Further computed tomography angiography (CTA) or magnetic resonance angiography (MRA) examinations were conducted and AC-LVO was identified. Among the 713 patients with AIS, 242 with prior stroke were excluded, and 471 patients with first-ever ischemic stroke (254 with disabling and 217 with non-disabling strokes) were included in the analysis for Model 2. For the external validation cohort, 150 eligible patients with AC-LVO were included in Model 1. Of the 99 patients with AIS, 82 who presented with the first episode were included in the analysis for model 2. The baseline characteristics of the included patients are presented in Tables 1, 2, and Supplementary Tables I–IV.

**Table 1:** Baseline characteristics of patients with anterior circulation large vessel occlusion.

	**Derivation cohort** **(*n* = 927)**	**Validation cohort** **(*n* = 150)**
Mean ± SD age, y	59.3 ± 12.4	62.0 ± 10.9
Males *n* (%)	626 (67.5)	108 (72.0)
Current smoking *n* (%)	268 (28.9)	57 (38.0)
Drinking *n* (%)	267 (28.8)	26 (17.3)
Hypertension *n* (%)	602 (64.9)	107 (71.3)
Coronary atherosclerotic heart	143 (15.4)	22 (14.7)
disease *n* (%)		
Atrial fibrillation *n* (%)	52 (5.6)	11 (7.3)
Diabetes *n* (%)	225 (24.3)	52 (34.7)
Hyperlipidaemia *n* (%)	292 (31.5)	14 (9.3)
Occluded vessels *n* (%)	442 (47.7)	51 (34.0)
Unilateral MCA		
Unilateral ICA	261 (28.2)	69 (46.0)
Multiple artery	224 (24.2)	30 (20.0)
BMI≥24 *n* (%)	795 (85.8)	108 (72.0)
Posterior circulation large vessel severe stenosis /occlusion n (%)	176 (19.0)	9 (6.0)
ApoB (g/L)	1.0 ± 0.3	0.7 ± 0.2
Homocysteine (μmol/L)	19.0 ± 11.2	15.9 ± 9.6
Acute ischemic stroke *n* (%)	713 (76.9)	99 (66.0)

*ApoB, apolipoprotein B; BMI, body mass index; ICA, internal carotid artery; IQR, interquartile range; MCA, middle cerebral artery; SD, standard deviation*.

**Table 2:** Baseline characteristics of patients with first-ever acute ischemic stroke (AIS) caused by anterior circulation large vessel occlusion.

	**Derivation cohort (*n* = 471)**	**Validation cohor (*n* = 82)**
Mean ± SD age, y	58.6 ± 12.7	61.0 ± 13.0
Males *n* (%)	321 (68.2)	59 (72.0)
Current smoking *n* (%)	147 (31.2)	35 (42.7)
Hypertension *n* (%)	290 (61.6)	51 (62.2)
Diabetes *n* (%)	105 (22.3)	25 (30.5)
Coronary atherosclerotic heart	69 (14.6)	7 (8.5)
disease *n* (%)		
Prior TIA *n* (%)	30 (6.4)	0 (0)
Hyperlipidaemia *n* (%)	158 (33.5)	8 (9.8)
BMI ≥24 *n* (%)	410 (87.0)	60 (73.2)
Median ASPECTS (IQR)	6 (3–8)	8 (7–9)
Occluded vessels *n* (%)	236 (50.1)	31 (37.8)
Unilateral MCA		
Unilateral ICA	133 (28.2)	36 (43.9)
Multiple artery	102 (21.7)	15 (18.3)
Anterior cerebral artery occlusion n (%)	87 (18.5)	3 (3.7)
Disabling ischemic stroke (NIHSS > 5) *n* (%)	254 (53.9)	31 (37.8)

*ASPECTS, Alberta Stroke Program Early CT Score; BMI, body mass index; ICA, internal carotid artery; IQR, interquartile range; MCA, middle cerebral artery; NIHSS, National Institutes of Health Stroke Scale; SD, standard deviation; TIA, transient ischemic attack*.

### Comparison Between the Models in the Derivation Cohort

**Figure 3:**
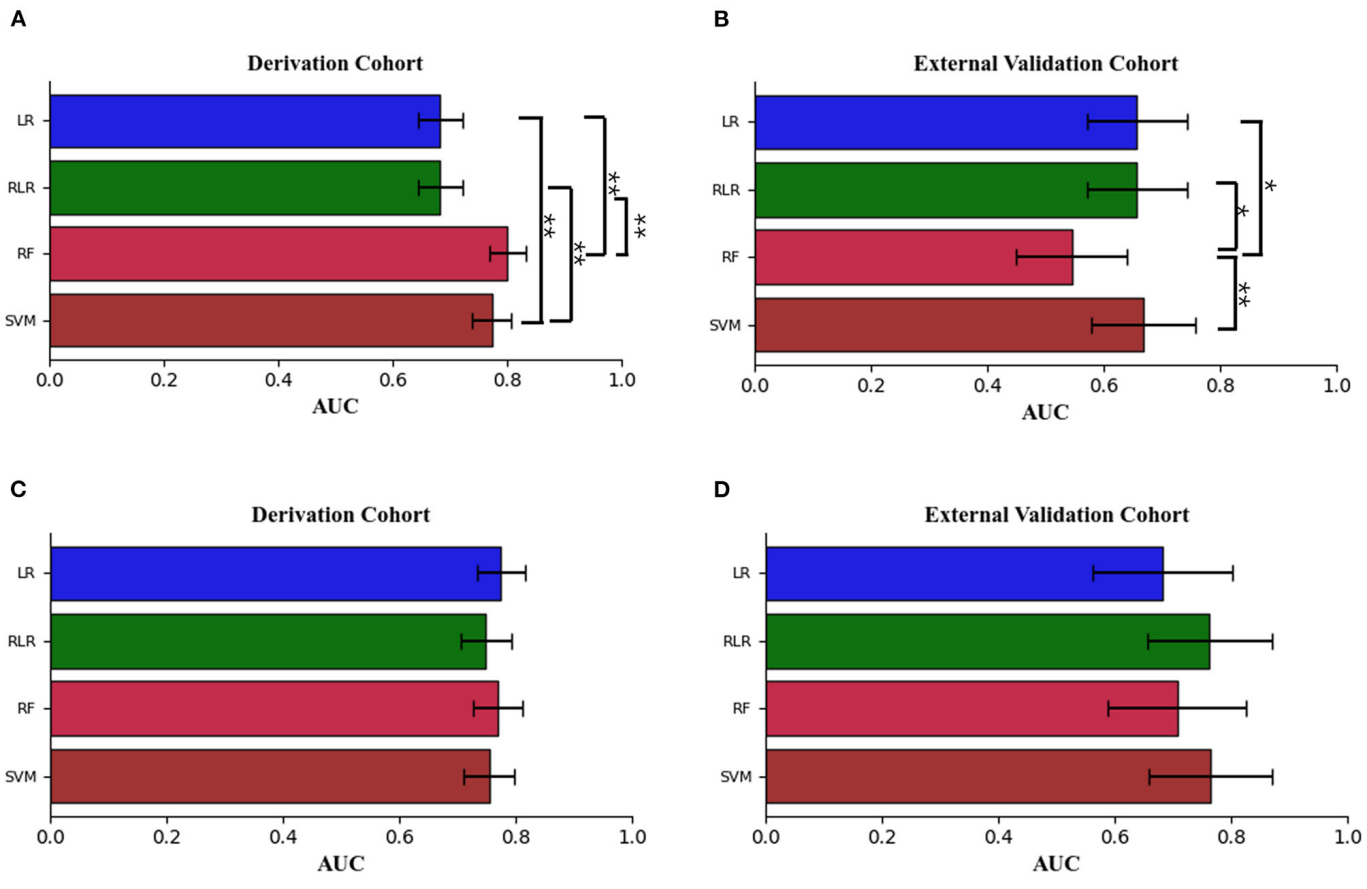
The means ± 95% CI of the receiver operating characteristic area under the curve (AUC) for models 1 and 2 are displayed as bar graphs using the derivation cohort data **(A,C)**, and the validation cohort data **(B,D)**. For the derivation cohort data, there were significant differences between random forest [RF], support vector machine [SVM] and logistic regression without regularization [LR], regularized logistic regression [RLR] in model 1. For the external validation cohort data, there were significant differences between the random forest [RF] and the other three machine learning methods in model 1. For the derivation and external validation cohort data, the Delong test with Bonferroni correction was used. LR indicates logistic regression without regularization; RF, random forest; RLR, regularized logistic regression; and SVM, support vector machine. *P < 0.01, **P < 0.001.

**Figure 4:**
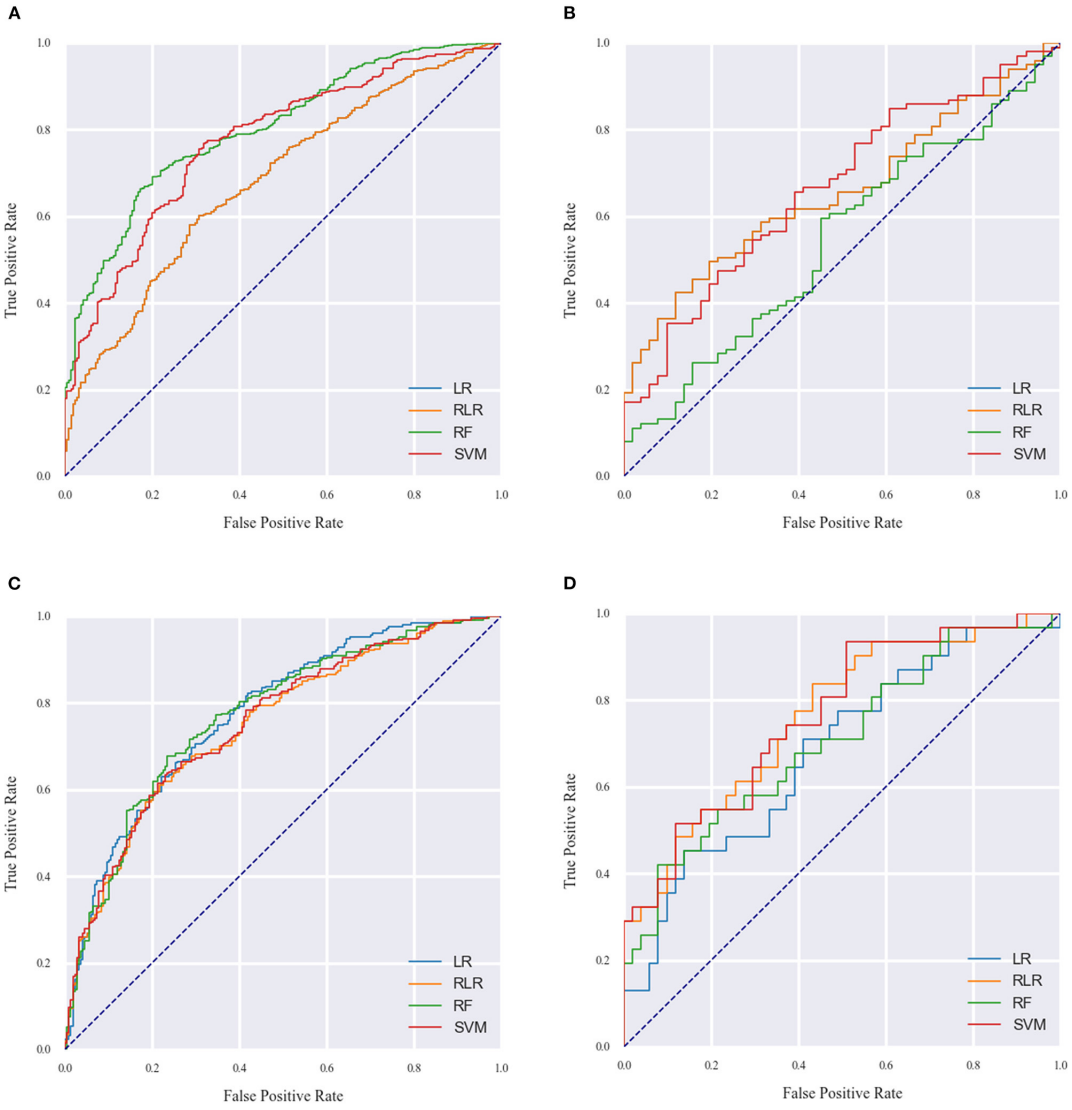
The AUC of the machine learning models for model 1 **(A,B)** and model 2 **(C,D)** on the derivation and external validation cohort data. LR indicates logistic regression without regularization; RF, random forest; RLR, regularized logistic regression, SVM, support vector machine.

The performance metrics of each approach for Models 1 and 2 in the derivation cohort are shown in Tables 3, 4, respectively. The receiver operating characteristic (ROC) curve (indicating the predictive performance of our LR/RLR/RF/SVM model) for each algorithm in the two models and the comparison among these machine learning algorithms are shown in **Figures 4A,C**. In model 1, the AUCs of RF and SVM were significantly higher than those of the LR and RLR, when using the DeLong test with Bonferroni correction (RF vs. LR, *P* < 0.0001; RF vs. RLR, *P* < 0.0001; SVM vs. LR, *P* < 0.0001; SVM vs. RLR, *P* < 0.0001; Figure 3A). Similar results were obtained for accuracy and F1-score. In model 2, while the differences in AUCs among the four machine learning algorithms were not significant (Figure 3C), the RF showed the most perfect classification accuracy (71.8%) compared to that of the other machine learning approaches.

**Table 3:** Scores for each algorithm of model 1 in derivation cohort.

**Model**	**AUC (95% CI)**	**PRC**	**Sensitivity**	**Specificity**	**Precision**	**NPV**	**Accuracy**	**F1_max_**
LR	0.68 (0.64–0.72)	0.88	57.9	71.5	87.1	33.8	61.1	0.87
RLR	0.68 (0.64–0.72)	0.88	57.9	71.5	87.1	33.8	61.1	0.87
RF	0.80 (0.77–0.83)	0.93	69.1	79.9	92.0	43.7	71.6	0.89
SVM	0.77 (0.74–0.81)	0.92	76.9	68.2	89.0	46.9	74.9	0.88

*AUC, receiver operator characteristic area under the curve; F1_max_, the maximum F1 score; LR, logistic regression without regularization; PRC, area under the precision-recall curve; RF, random forest; RLR, regularized logistic regression; SVM, support vector machine; NPV, negative predictive value*.

**Table 4:** Scores for each algorithm of model 2 in derivation cohort.

**Model**	**AUC (95% CI)**	**PRC**	**Sensitivity**	**Specificity**	**Precision**	**NPV**	**Accuracy**	**F1_max_**
LR	0.78 (0.74–0.81)	0.78	63.0	77.9	76.9	64.3	69.9	0.76
RLR	0.75 (0.71–0.79)	0.77	61.0	78.3	76.7	63.2	69.0	0.74
RF	0.77 (0.73–0.81)	0.78	67.7	76.5	77.1	66.9	71.8	0.75
SVM	0.76 (0.71–0.80)	0.78	63.4	77.0	76.3	64.2	69.6	0.74

*AUC, receiver operator characteristic area under the curve; F1_max_, the maximum F1 score; LR, logistic regression without regularization; PRC, area under the precision-recall curve; RF, random forest; RLR, regularized logistic regression; SVM, support vector machine; NPV, negative predictive value*.

### Comparison Between the Models in the External Validation Cohort

The ROC curves for Models 1 and 2 in the external validation cohort are shown in Figures 4B,D. In Model 1, RF exhibited the worst performance among the machine learning models (Table 5). The AUCs in LR, RLR and SVM were significantly higher than that in RF, when using the Delong test with Bonferroni correction (LR vs. RF, *P* = 0.0048; RLR vs. RF, *P* = 0.0048, SVM vs. RF, *P* = 0.0006; Figure 3B). In Model 2, there was no difference in AUCs among the four machine learning algorithms (Figure 3D). The AUC of each algorithm was as follows: LR 0.68 (95% CI 0.56–0.8), RLR 0.76 (95% CI 0.66–0.87), RF 0.71 (95% CI 0.59–0.83) and SVM 0.77 (95% CI 0.66–0.87) (Table 6).

**Table 5:** Scores for each algorithm of model 1 in external validation cohort.

**Model**	**AUC (95% CI)**	**PRC**	**Sensitivity**	**Specificity**	**Precision**	**NPV**	**Accuracy**	**F1_max_**
LR	0.66 (0.57–0.74)	0.82	42.4	88.2	87.5	44.1	58.0	0.80
RLR	0.66 (0.57–0.74)	0.82	42.4	88.2	87.5	44.1	58.0	0.80
RF	0.55 (0.45–0.64)	0.72	59.6	54.9	72.0	41.2	58.0	0.80
SVM	0.67 (0.58–0.76)	0.81	65.7	60.8	76.5	47.7	64.0	0.80

*AUC, receiver operator characteristic area under the curve; F1_max_, the maximum F1 score; LR, logistic regression without regularization; PRC, area under the precision-recall curve; RF, random forest; RLR, regularized logistic regression; SVM, support vector machine; NPV, negative predictive value*.

**Table 6:** Scores for each algorithm of model 2 in external validation cohort.

**Model**	**AUC (95% CI)**	**PRC**	**Sensitivity**	**Specificity**	**Precision**	**NPV**	**Accuracy**	**F1_max_**
LR	0.68 (0.56–0.80)	0.60	45.2	86.3	66.7	72.1	70.7	0.60
RLR	0.76 (0.66–0.87)	0.71	83.9	56.9	54.2	85.3	67.1	0.66
RF	0.71 (0.59–0.83)	0.65	41.9	92.2	76.5	72.3	73.2	0.61
SVM	0.77 (0.66–0.87)	0.71	93.5	49.0	52.7	92.6	65.9	0.67

*AUC, receiver operator characteristic area under the curve; F1_max_, the maximum F1 score; LR, logistic regression without regularization; PRC, area under the precision-recall curve; RF, random forest; RLR, regularized logistic regression; SVM, support vector machine; NPV, negative predictive value*.

### Important Variables of the Machine Learning Models

**Figure 5:**
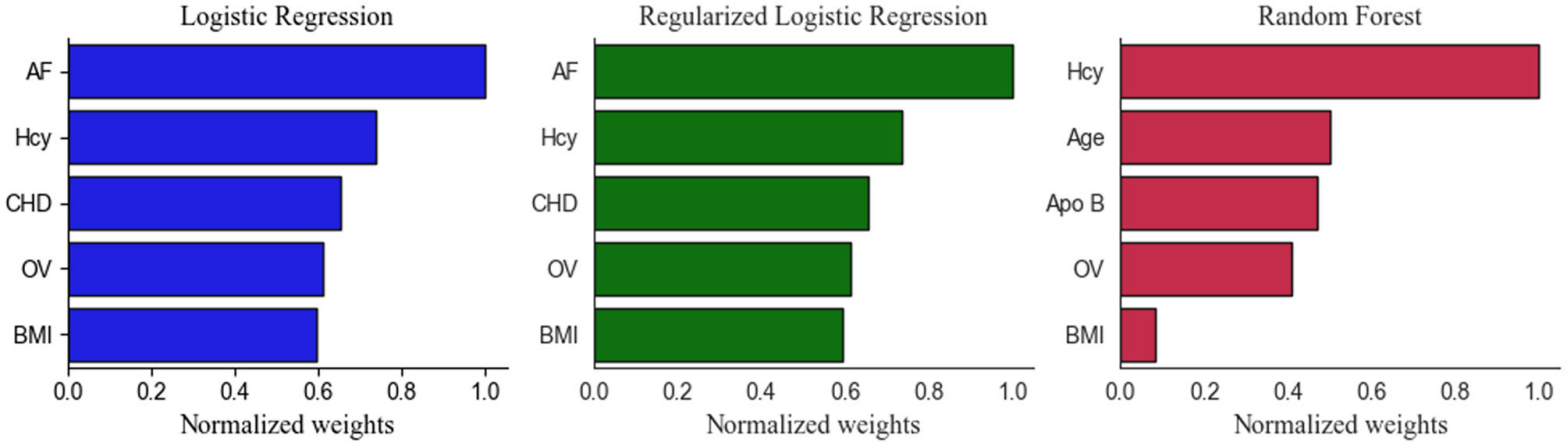
Top five Important Features in the Model 1. Apo B indicates apolipoprotein B; AF, atrial fibrillation; BMI, body mass index; CHD, coronary atherosclerotic heart disease; Hcy, homocysteine; LR, logistic regression without regularization; OV, occluded vessels; RF, random forest; RLR, regularized logistic regression.

**Figure 6:**
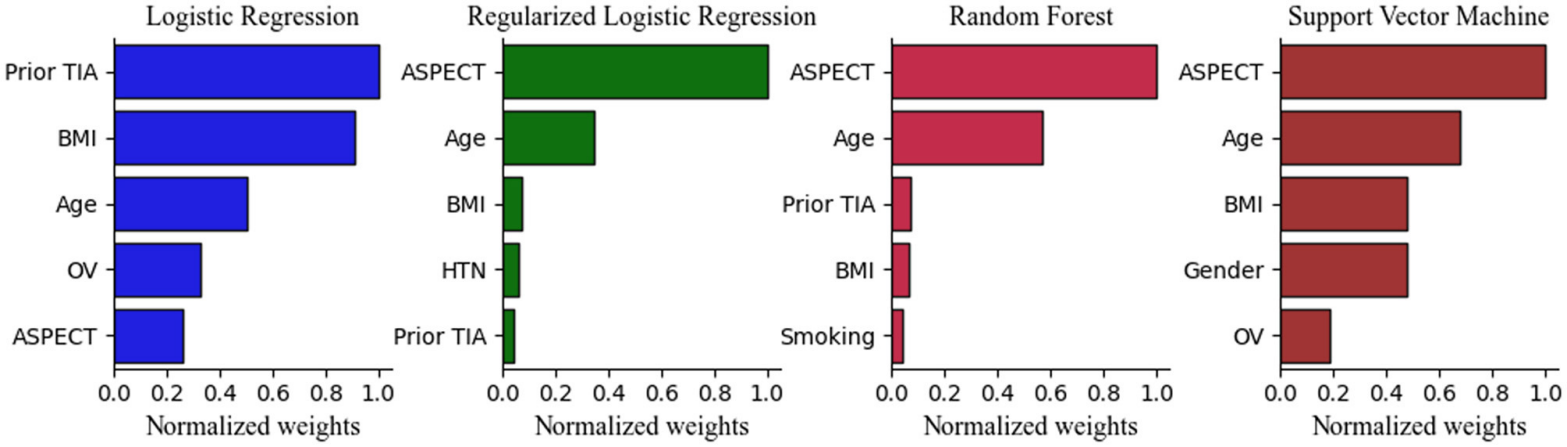
Top 5 Important Features in the Model 2. ASPECT indicates Alberta Stroke Program Early CT Score; BMI, body mass index; HTN, hypertension; LR, logistic regression without regularization; OV, occluded vessels; RF, random forest; RLR, regularized logistic regression; SVM, support vector machine; TIA, transient ischemic attack.

After calculating the importance of each feature, the top five selected variables of Models 1 and 2 were ranked by their discriminative performance (Figures 5, 6). For LR and RLR, the absolute value of the standardized regression coefficient was calculated in both models. For RF, the important features for information gain and Gini coefficient were ranked in Models 1 and 2 respectively. For SVM, the absolute value of the weight was used to rank the variables only in model 2 due to the introduction of the kernel function in Model 1. The absolute values of the important metrics for the features were normalized, ensuring the comparability in feature importance ranking. In Model 1, homocysteine, occluded vessels and BMI appeared together in the top five rankings of all machine learning algorithms. In addition, coronary atherosclerotic heart disease was an important feature in both LR and RLR. Age and Apo B appeared to be important variables in RF. In Model 2, ASPECT, age and BMI were common variables for all machine learning algorithms. Prior TIA was included in LR, RLR, and RF. Hypertension, current smoking, and gender appeared in RLR, RF, and SVM, respectively. Furthermore, occluded vessels coexisted in LR and SVM.

## Discussion

This study demonstrated that the use of a machine learning approach can predict the risk of AIS and severity of ischemic stroke in AC-LVO from clinical data. To the best of our knowledge, this is the first report on an attempt to predict AIS and severity of neurological impairment in patients with AC-LVO using the machine learning approach. The machine learning algorithm can eliminate linearity and has various ways of overcoming the imperfections of the polyfactorial models such as overfitting of models and collinearity of variables, which may lead to a series of problems when it comes to variable selection (17). In the two prediction models in this study, 14 and 12 common variables were collected, respectively, bypassing the traditional method of variable selection.

Contrary to the findings in the derivation cohort of model 1 that RF showed significantly better predictive performance than LR and RLR, in the validation cohort, RF had the worst performance among the machine learning models. The decision trees of RF forced interactions between the features, which might make the result rather inferior if the majority of the features have no or very weak interactions. Therefore, we suspect that the RF was not able to carry on an accurate classified forecast owing to extremely weak interactions between the variables in our dataset. Moreover, the small data sets with 150 cases in the validation cohort may be another reason for the poor performance of the RF. In model 2, although the LR showed a predictive property similar to those of the other three algorithms both in the validation cohort and derivation cohort, the RLR exhibited a higher AUC compared with LR in the validation cohort, this was as a result of the poor generalization performance of LR compared with other algorithms. Accordingly, LR with L2 regularization was implemented in this study to avoid overfitting and improve the generalization performance and robustness of the model; thus, a more optimal result was obtained with an AUC of 0.76.

As shown in Figures 5, 6, the important features were not entirely consistent in the machine learning algorithms in model 1 and model 2. As important variables of model 1, homocysteine, BMI, and occluded vessels (unilateral MCA) appeared in all three algorithms, and atrial fibrillation and coronary atherosclerotic heart disease were detected in both LR and RLR. Elevated blood homocysteine concentration increases the risk of ischemic stroke by inducing oxidative damage to vascular endothelial cells and enhancing platelet adhesion to endothelial cells, especially in large vessel strokes (18–22). The results of our study are in accordance with the aforementioned studies, suggesting that elevated homocysteine levels may be a significant marker for predicting ischemic stroke in AC-LVO.

Regarding the association between BMI and ischemic stroke, a previous meta-analysis revealed a J-shaped dose-response relationship between being overweight or obese and an increased risk of incident ischemic stroke (23). However, few studies have focused on the relationship between BMI and risks of ischemic stroke subtype (24, 25). Our study showed a robust positive association between overweight/obesity and AC-LVO AIS. Possible explanations for our findings include insulin resistance, endothelial dysfunction, and inflammation, which have been considered to influence the relationship between obesity and atherosclerosis (26). Moreover, our findings further revealed that a high BMI (≥ 24 kg/m^2^) shows a greater predisposes to disabling than non-disabling ischemic stroke with AC-LVO, emphasizing the importance of weight control and aerobic fitness.

The compensation of the collateral pathway in MCA occlusion mainly depends on the pia meningeal branch from the anterior cerebral artery and the posterior cerebral artery with worse compensatory ability than the circle of Willis, which means it would result in hemodynamic failure and is more prone to decompensation (27). Our study delves deeper into this field and demonstrates that unilateral MCA occlusion plays a crucial role in the occurrence of ischemic stroke. Furthermore, we found that stroke severity at admission was greater in the multiple AC-LAO patients than in unilateral MCA occlusion or unilateral ICA occlusion patients. This is consistent with a previously published study of patients with AC-LVO AIS, which showed that high NIHSS was associated with multiple AC-LAO (28).

Cardioembolism might be responsible for large vessel occlusion, in which atrial fibrillation accounts for ~50% (29, 30). Atrial fibrillation is strongly associated with a high occurrence rate of LVO, suggesting that it may be a potential risk factor for LVO (31). Otherwise, large emboli that block intracranial vessels usually originate from the left atrial appendage in patients with symptomatic carotid stenosis or atrial fibrillation (32). Similarly, in our analysis, atrial fibrillation showed a robust association with AC-LVO AIS, further suggesting that knowledge of the potential complications of atrial fibrillation is likely to motivate both patient and clinician to comply with standard treatment.

Large-artery atherosclerotic stroke is associated with a high risk of coronary atherosclerotic heart disease (33). Nevertheless, our results indicate that coronary atherosclerotic heart disease is associated with a low risk of AIS in AC-LVO patients. One explanation for this finding might be that coronary atherosclerosis is significantly correlated with stenosis of the extracranial carotid; therefore, the development of intracranial anterior circulation large vessel occlusion may be independent of coronary atherosclerotic heart disease (34). Furthermore, antiplatelet and statin therapy in coronary atherosclerotic heart disease may reduce the risk of ischemic stroke in AC-LVO.

Apolipoprotein B is the primary apolipoprotein component of chylomicrons and low-density lipoproteins (35). In this study, we found that elevated serum levels of Apo B were associated with an increased risk of ischemic stroke in AC-LVO. Additionally, a Mendelian randomization study reported a positive correlation of Apo B with large artery stroke and small vessel stroke (36). Therefore, we advocate Apo B as a marker of routine serum lipid examination.

In our study, age emerged as an important predictor in both models, as well as in a previously developed model for predicting the clinical outcome of AIS with LVO (17). In general, our results indicate that the prevalence of ischemic stroke and disability increases with age in patients with AC-LVO. In addition, our data also suggested that ASPECT was the common element included in all machine learning methods. Studies have demonstrated that diffusion-weighted imaging (DWI) ASPECTS which represents infarct volume, is a significant independent predictor of functional outcome in AC-LVO strokes (37). Correspondingly, patients presenting with ASPECTS ≥7 are correlated with favorable outcomes following intravascular or thrombolytic therapy (38, 39). Our study further supports the association between ASPECT and the severity of neurological defects in first-ever ischemic stroke with AC-LVO. Consequently, a lower score of ASPECTS suggests less preserved brain parenchyma and predicts severe neurological impairment in patients with first-ever AC-LVO ischemic strokes.

It is well established that TIA increases the risk of ischemic stroke. In the present study, we found that prior TIA decreased ischemic stroke severity at admission, which is similar to the results of Marc Gotkine et al. showing that previous TIA was independently associated with lower severity of the ischemic stroke and a better short-term outcome (40). Prior TIA may have a neuroprotective effect on the subsequent ischemic stroke.

The chief strength of this study is the development and external validation of a new scoring tool, which predicts the risk of ischemic stroke and the severity of ischemia in AC-LVO based on machine learning approaches. Nevertheless, this study has several limitations. Foremost, few neuroimaging features were taken into consideration, excluding others such as the collateral flow status which might improve the predictive performance of the models. Further evaluation of the level of collateral circulation is necessary. Second, the sample size for this study was small which might have been due to the stringent inclusion criteria for patients with AC-LVO. As a result, the performance advantages of machine learning models may not have been fully realized. Finally, this was a retrospective study; the performance of the model needs to be tested in a prospective population in future studies.

## Data Availability Statement

The raw data supporting the conclusions of this article will be made available by the authors, without undue reservation.

## Ethics Statement

The studies involving human participants were reviewed and approved by Research Ethics Committee of the Second Hospital of Hebei Medical University on June 29, 2021 (approval No. 2021-R435). Written informed consent for participation was not required for this study in accordance with the national legislation and the institutional requirements.

## Author Contributions

JC and JY drafted and revised the manuscript, participated in the study conception and design, performed the statistical analyses, and analyzed and interpreted the data. XLiu participated in the conception and design of the study, data interpretation, and made a major contribution to manuscript revision. JY assisted in designing the machine learning model and in data analysis. KZ, GX, RZ, XLi, LL, YZ, LZ, PY, LX, TL, JT, PZ, SY, QW, and LG participated in the design of the study and contributed to manuscript revision. All authors contributed to the article and approved the submitted version.

## Funding

This study was supported by a grant from XLiu from the National Natural Science Foundation of China (81571160).

## Conflict of Interest

The authors declare that the research was conducted in the absence of any commercial or financial relationships that could be construed as a potential conflict of interest.

## Publisher's Note

All claims expressed in this article are solely those of the authors and do not necessarily represent those of their affiliated organizations, or those of the publisher, the editors and the reviewers. Any product that may be evaluated in this article, or claim that may be made by its manufacturer, is not guaranteed or endorsed by the publisher.
