# Supplementary material for: Machine Learning-Based Model for Predicting Incidence and Severity of Acute Ischemic Stroke in Anterior Circulation Large Vessel Occlusion
Source: Front Neurol. 2021 Dec 2;12:749599. doi: 10.3389/fneur.2021.749599 (PMC8675605; doi:10.3389/fneur.2021.749599)
Supplement: Supplementary file 1 [file Data_Sheet_1.doc]

**Supplement**

**Table I** Baseline Characteristics of Patients in derivation cohort for model 1

|  | AIS related with AC- LVO (n=713) | Asymptomatic AC-LVO (n=214) | *P*-value |
| --- | --- | --- | --- |
| Mean±SD age, y | 59.5±12.1 | 58.4±13.1 | 0.256 |
| Males n (%) | 481 (67.5) | 145 (67.8) | 0.935 |
| Current smoking n (%) | 210 (29.5) | 58 (27.1) | 0.506 |
| Drinking n (%) | 215 (30.2) | 52 (24.3) | 0.097 |
| Hypertension n (%) | 468 (65.6) | 134 (62.6) | 0.417 |
| Coronary atherosclerotic heart disease n (%) | 102 (14.3) | 41 (19.2) | 0.085 |
| Atrial fibrillation n (%) | 45 (6.3) | 7 (3.3) | 0.09 |
| Diabetes n (%) | 179 (25.1) | 46 (21.5) | 0.280 |
| Hyperlipidaemia n (%) | 221 (31.0) | 71 (33.2) | 0.547 |
| Occluded_vessels n (%) | | | < 0.0001 |
| Unilateral MCA | 360 (50.5) | 82 (38.3) |  |
| Unilateral ICA | 171 (24.0) | 90 (42.1) |  |
| Multiple artery | 182 (25.5) | 42 (19.6) |  |
| BMI≥24 n (%) | 620 (87.0) | 175 (81.8) | 0.057 |
| Posterior circulation large vessel severe stenosis /occlusion n (%) | 145 (20.3) | 31 (14.5) | 0.056 |
| ApoB | 1.0±0.3 | 0.9±0.3 | 0.074 |
| Homocysteine | 19.8±12.4 | 15.9±4.6 | < 0.0001 |

AC- LVO, anterior circulation large vessel occlusion; AIS, acute ischemic stroke; ApoB, apolipoprotein B; BMI, body mass index; ICA, internal carotid artery; IQR, interquartile range; MCA, middle cerebral artery; SD, standard deviation

**Table II** Baseline Characteristics of Patients in external validation cohort for model 1

|  | AIS related with AC- LVO (n=99) | Asymptomatic AC-LVO (n=51) | *P*-value |
| --- | --- | --- | --- |
| Mean±SD age, y | 61.1±12.2 | 64.3±7.3 | 0.043 |
| Males n (%) | 73 (73.7) | 35 (68.6) | 0.509 |
| Current smoking n (%) | 41 (41.4) | 16 (31.4) | 0.230 |
| Drinking n (%) | 23 (23.2) | 3 (5.9) | 0.008 |
| Hypertension n (%) | 65 (65.7) | 42 (82.4) | 0.032 |
| Coronary atherosclerotic heart disease n (%) | 10 (10.1) | 12 (23.5) | 0.028 |
| Atrial fibrillation n (%) | 9 (9.1) | 2 (3.9) | 0.334 |
| Diabetes n (%) | 35 (35.4) | 17 (33.3) | 0.280 |
| Hyperlipidaemia n (%) | 10 (10.1) | 4 (7.8) | 0.773 |
| Occluded_vessels n (%) | | | 0.983 |
| Unilateral MCA | 34 (34.3) | 17 (33.3) |  |
| Unilateral ICA | 45 (45.5) | 24 (47.1) |  |
| Multiple artery | 20 (20.2) | 10 (19.6) |  |
| BMI≥24 n (%) | 72 (72.7) | 36 (70.6) | 0.782 |
| Posterior circulation large vessel severe stenosis /occlusion n (%) | 6 (6.1) | 3 (5.9) | 1.0 |
| ApoB | 0.7±0.2 | 0.7±0.2 | 0.648 |
| Homocysteine | 15.3（11.5-19.6） | 11.3（8.4-15.3） | < 0.0001 |

AC- LVO, anterior circulation large vessel occlusion; AIS, acute ischemic stroke; ApoB, apolipoprotein B; BMI, body mass index; ICA, internal carotid artery; IQR, interquartile range; MCA, middle cerebral artery; SD, standard deviation

**Table III** Baseline Characteristics of Patients in derivation cohort for model 2

| Characteristic | Disabling ischemic stroke (n=254) | Non-disabling ischemic stroke (n=217) | *P*-value |
| --- | --- | --- | --- |
| Mean ± SD age, y | 61.7±11.2 | 54.9±12.5 | < 0.0001 |
| Males n (%) | 172 (67.7) | 149 (68.7) | 0.826 |
| Current smoking n (%) | 72 (28.3) | 75 (34.6) | 0.147 |
| Hypertension n (%) | 164 (64.6) | 126 (58.1) | 0.148 |
| Diabetes n (%) | 53 (20.9) | 52 (24.0) | 0.421 |
| Coronary atherosclerotic heart disease n (%) | 43 (16.9) | 26 (12.0) | 0.130 |
| Prior TIA n (%) | 6 (3.4) | 24(11.1) | < 0.0001 |
| Hyperlipidaemia n (%) | 83 (32.7) | 75 (34.6) | 0.666 |
| BMI ≥24 n (%) | 235 (92.5) | 175 (80.6) | < 0.0001 |
| Median ASPECTS (IQR) | 4（2-7） | 7（5-8） | < 0.0001 |
| Occluded vessels n (%) | | | 0.034 |
| Unilateral MCA | 124 (48.8) | 112 (51.6) |  |
| Unilateral ICA | 64 (25.2) | 69 (31.8) |  |
| Multiple artery | 66 (26.0) | 36 (16.6) |  |
| Anterior cerebral artery occlusion n (%) | 53 (20.9) | 34 (15.7) | 0.147 |

ASPECTS, Alberta Stroke Program Early CT Score; BMI, body mass index; ICA, internal carotid artery; IQR, interquartile range; MCA, middle cerebral artery; SD, standard deviation; TIA, transient ischemic attack.

**Table IV** Baseline Characteristics of Patients in external validation cohort for model 2

| Characteristic | Disabling ischemic stroke (n=31) | Non-disabling ischemic stroke (n=51) | *P*-value |
| --- | --- | --- | --- |
| Mean ± SD age, y | 60.6±14.3 | 61.2±12.3 | 0.845 |
| Males n (%) | 21 (67.7) | 38 (74.5) | 0.508 |
| Current smoking n (%) | 13 (41.9) | 22 (43.1) | 0.915 |
| Hypertension n (%) | 164 (64.5) | 126 (60.8) | 0.735 |
| Diabetes n (%) | 8 (25.8) | 17 (33.3) | 0.473 |
| Coronary atherosclerotic heart disease n (%) | 3 (9.7) | 4 (7.8) | 1.0 |
| Prior TIA n (%) | 0 (0) | 0 (0) | N |
| Hyperlipidaemia n (%) | 1 (3.2) | 7 (13.7) | 0.248 |
| BMI ≥24 n (%) | 24 (77.4) | 36 (70.6) | 0.498 |
| Median ASPECTS (IQR) | 7（5-8） | 8（8-9） | < 0.0001 |
| Occluded vessels n (%) | | | 0.106 |
| Unilateral MCA | 15 (48.4) | 16 (31.4) |  |
| Unilateral ICA | 9 (29.0) | 27 (52.9) |  |
| Multiple artery | 7 (22.6) | 8 (15.7) |  |
| Anterior cerebral artery occlusion n (%) | 1 (3.2) | 2 (3.9) | 1.0 |

ASPECTS, Alberta Stroke Program Early CT Score; BMI, body mass index; ICA, internal carotid artery; IQR, interquartile range; MCA, middle cerebral artery; SD, standard deviation; TIA, transient ischemic attack.

**Figure I**


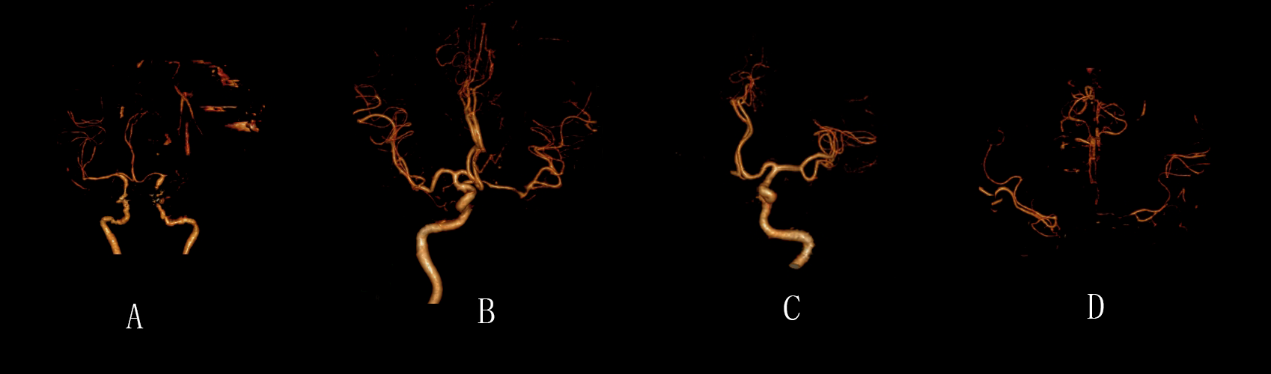


Figure I. Anterior circulation LAO types: (A) unilateral MCA, (B) unilateral ICA, (C) multiple artery: tandem ICA/MCA, (D) multiple artery: bilateral ICA
